# Breaking Barriers with Sound: The Implementation of Histotripsy in Cancer

**DOI:** 10.3390/cancers17152548

**Published:** 2025-08-01

**Authors:** Ashutosh P. Raman, Parker L. Kotlarz, Alexis E. Giff, Katherine A. Goundry, Paul Laeseke, Erica M. Knavel Koepsel, Mosa Alhamami, Dania Daye

**Affiliations:** 1 Harvard Medical School, Boston, MA 02115, USAalexis_giff@hms.harvard.edu (A.E.G.); katherine_goundry@hms.harvard.edu (K.A.G.); ddaye@mgh.harvard.edu (D.D.); 2 Harvard-MIT Division of Health Sciences and Technology, Boston, MA 02115, USA; 3 Athinoula A. Martinos Center for Biomedical Imaging, Massachusetts General Hospital, Charlestown, MA 02129, USA; 4 Department of Radiology, University of Wisconsin, Madison, WI 53705, USA; plaeseke@wisc.edu (P.L.); knavel@wisc.edu (E.M.K.K.); alhamami@wisc.edu (M.A.); 5 Department of Medical Physics, University of Wisconsin, Madison, WI 53705, USA; 6 Department of Radiology, Massachusetts General Hospital, Boston, MA 02114, USA

**Keywords:** histotripsy, ultrasound, interventional radiology, ablation, hepatocellular carcinoma, metastatic colorectal carcinoma, immunomodulation, real-time feedback, non-invasive, cavitation

## Abstract

Histotripsy is a novel, noninvasive, non-thermal technology invented in 2004 for the precise destruction of biologic tissue. It offers a powerful alternative to more conventional thermal or surgical interventions. Using short-pulse, low-duty cycle ultrasonic waves, histotripsy creates cavitation bubble clouds that selectively and precisely destroy targeted tissue in a predefined volume while sparing critical structures like bile ducts, ureters, and blood vessels. Such precision is of value when treating tumors near vital structures. The FDA has cleared histotripsy for the treatment of all liver tumors. Major medical centers are currently spearheading clinical trials, and some institutions have already integrated the technology into patient care. Histotripsy is now being studied for a host of other cancers, including primary kidney and pancreatic tumors. Preclinical murine and porcine models have already revealed promising outcomes. One of histotripsy’s primary advantages is its non-thermal mechanical actuation. This feature allows it to circumvent the limitations of heat-based techniques, including the heat sink effect and unpredictable treatment margins near sensitive tissues. In addition to its non-invasive ablative capacities, it is being preliminarily explored for its potential to induce immunomodulation and promote abscopal inhibition of distant, untreated tumors through CD8+ T cell responses. Thus, it may provide a multilayered therapeutic effect in the treatment of cancer. Histotripsy has the potential to improve precision and outcomes across a multitude of specialties, from oncology to cardiovascular medicine. Continued trials are crucial to further expand its applications and validate its long-term efficacy. Due to the speed of recent developments, the goal of this review is to provide a comprehensive and updated overview of histotripsy. It will explore its physics-based mechanisms, differentiating it from similar technologies, discuss its clinical applications, and examine its advantages, limitations, and future.

## Simple Summary

Histotripsy is a novel surgical technology that involves utilization of rapid and strong ultrasound waves to precisely destroy cancer tissue, primarily in the liver but also increasingly in other organs. Histotripsy is being progressively adopted in surgical settings due to its advantages over traditional methods. This review describes histotripsy in depth, from its clinical niche and technical foundations to research and clinical trials currently taking place. Importantly, the current implementation of histotripsy is comprehensively presented in a manner that informs readers about the typical patient experience, as well as other important factors to the clinical employment of the technology. Through this review, we hope to compile and present histotripsy in an understandable manner, to inform clinicians, health systems, researchers, and patients alike on the current state of the technology.

## 1. Introduction

The treatment landscape for solid tumor malignancies has rapidly evolved in recent years, with surgical resection, fractionated radiation, and standard chemotherapy currently established as the mainstays of tumor destruction. Nonetheless, the achievement of clean resection margins and lasting remissions remains an issue; five-year survival rates for abdominal cancers like hepatocellular carcinoma (HCC), intrahepatic cholangiocarcinoma (ICC), pancreatic adenocarcinoma (PAC) and late-stage renal cell carcinoma (RCC) still hover around 10–50% [1,2,3]. Potential reasons for this abysmal survival rate include incomplete tumor resection, tumor inaccessibility due to proximity to vital structures, high incidence of recurrence or metastasis, and immune shielding of the tumor environment [4,5,6,7,8,9].

With the advent of immunotherapy and targeted therapy solutions, these survival rates are steadily improving. Another valuable approach to tumor treatment is minimally invasive surgery; laparoscopic and robotic-assisted procedures have been increasingly adopted in modern surgery due to their reduced intra- and post-operative complications and enhanced precision. However, despite advances, these types of surgical resection still require significant refinement to enhance combinatorial tumor clearance and to significantly debulk tumor mass, while also minimizing treatment-associated complications like thrombosis, recurrence, and unintended iatrogenic damage of nearby tissue [10].

Currently utilized minimally invasive approaches in interventional radiology include transarterial embolization, and thermal ablation, like high-intensity focused ultrasound (HIFU), radiofrequency ablation and microwave ablation [11,12,13]. While these methods offer multifaceted aid in tumor management, they have some disadvantages including intraprocedural radiation exposure, imprecision, deleterious systemic effects, inconsistent treatment efficacy, and an inability to concurrently visualize treatment course [14,15,16,17]. Thermal ablation, including HIFU, is susceptible to a heat-sink effect in the treatment zone of highly perfused tissues, making it difficult to accurately treat near large vessels [18].

Histotripsy, a term derived from Greek that means “to break down soft tissue,” presents a novel paradigm shift in the ablation of solid tumors. It is a non-thermal, non-ionizing, noninvasive technique that utilizes short-pulsed ultrasound waves, with the option of real-time B-mode ultrasound imaging feedback, to mechanically disrupt tissue through cavitation clouds. Part of histotripsy’s appeal is its ability to destroy tissue in a precise manner while sparing nearby collagen-rich vessels and ducts [19,20]. Its noninvasive approach, lack of thermal energy-based actuation, and integration with real-time imaging feedback address many of the issues of current therapeutic techniques [21]. Additionally, histotripsy has been shown experimentally to work synergistically with immunotherapy and chemotherapy to elicit broad anti-tumor immune reactions, thereby meaningfully reducing the likelihood of recurrence following treatment [22]. Histotripsy was first demonstrated in 2004 at the University of Michigan; human trials were conducted thereafter, in collaboration with the University of Wisconsin and University of Michigan, across six other sites in the United States and six sites in Europe, during the #HOPE4LIVER clinical trial in 2021 [23,24]. Recently, the FDA approved the usage of the HistoSonics Inc. (Plymouth, MN, USA) Edison platform for the treatment of primary and metastatic liver tumors. With this approval, histotripsy is now being assessed on a larger scale for safety and efficacy, achieving promising initial evaluations [25]. Histotripsy is quickly emerging as a high-value tool in the oncologic space, with more institutions adopting its use and trials for other organs underway.

This review aims to synthesize current preclinical and clinical research, technical documentation, and current implementation protocols to provide a comprehensive understanding of histotripsy. By addressing gaps in the current literature and building understanding on how histotripsy beneficially differs from other ablation techniques, we hope to inform clinicians on how histotripsy can potentially augment their clinical practice. The following sections will consolidate the technical aspects of histotripsy, as well as recent animal models and clinical trials utilizing histotripsy in the treatment of solid tumors. The latter sections will focus on the current clinical implementation of histotripsy, the typical patient treatment journey, concurrent imaging methods, and treatment monitoring—particularly in light of the recent FDA approval for non-research clinical usage. Finally, we discuss emerging applications, complications, limitations, and future directions for this promising technology.

## 2. Technical Background for Clinicians

Histotripsy relies on cavitation, a process where pulsed ultrasound waves generate peak negative pressures that exceed the intrinsic threshold of biological tissues, leading to the formation of a dense bubble cloud within the targeted tissue [26]. Cavitation begins when high amplitude pulses create negative pressure that overcome the surface tension of preexisting gas pockets which are roughly 2–5 nm in diameter. These gas pockets are thought to be formed from dissolved atmospheric gas within tissue fluids and crevices found in vessel walls and intracellularly. The gas pockets expand in the setting of negative pressure forming bubble clouds which consist of atmospheric gases in the focal zone of the ultrasound [26]. The gas pockets allow the bubbles to form in the focal zone of the ultrasound. There is a threshold for the initiation of a cavitation bubble, and it varies based on the tissue composition. The threshold is typically 26–30 MPa in water-based tissues like blood clots, liver, kidney, heart, brain, spleen, and pancreas and around 14–17 MPa in fat-based tissues as a result of mechanical and acoustic properties. Different models have shown that tissue density, attenuation, dynamic viscosity, surface tension, and elastic (Young’s) modulus all play a role in cavitation threshold [27,28,29]. Histotripsy as a technique allows for targeted cavitation by modulating pressure amplitude and pulse parameters. The specificity of this technique allows for selective tissue disruption while keeping unintended side effects towards adjacent structures to a minimum [26,29].

Histotripsy uses two primary methods to initiate cavitation bubble clouds: intrinsic threshold histotripsy and shock-scattering histotripsy. Intrinsic threshold histotripsy uses short ultrasound pulses of 1–2 cycles to generate a negative pressure phase. When the phase surpasses the intrinsic cavitation threshold of the tissue, bubbles will form and propagate out from the transducer in a linear pattern [26]. Shock-scattering histotripsy uses longer pulses of 3–10 cycles to create high frequency (500 kHz–3 MHz) harmonic waves from nonlinear acoustic propagation. The waves create a compressed positive phase followed by an inverted negative phase that travels back towards the transducer. This creates a fan-shaped bubble cloud for cavitation. The nonlinear propagation makes it so that peak negative pressure may be lowered (~15 MPa) offering an advantage in tissue disruption [30]. Each mechanism offers distinct spatiotemporal characteristics suited to different clinical applications. A comparison of key acoustic parameters across these modalities is provided in Table 1.

Once cavitation is initiated, the bubbles within the cloud undergo rapid cycles of expansion and collapse. These cycles create intense mechanical stress that disrupts the cell membranes as well as extracellular matrix (ECM) components. During the expansion phase, the negative pressures generated allow the bubbles to expand to a maximum volume from 2 to 5 nm to greater than 100 μm [36]. This is followed by a collapse due to the surrounding pressures that generate localized shockwaves that cause mechanical fragmentation of tissue, typically within a few hundred microseconds [37]. Repeated cycles of expansion and collapse create a cumulative mechanical strain on adjacent cells, breaking down targeted tissue structures. This is known as mechanical fractionation, allowing histotripsy to ablate targeted tissues without injuring collateral structures. This mechanical fractionation requires the cyclic strain from multiple pulses [38]. The ability to regulate bubble cloud parameters ensures preservation of healthy tissue, which makes histotripsy an optimal technique for noninvasive tissue destruction for applications in ablation that precisely maintains organ and vascular structure and function [31].

The cavitation area determines the treatment zone which makes it important to control ultrasound parameters like pulse repetition frequency (PRF) and pulse duration to achieve predictable and reproducible outcomes [31]. The ultrasound transducer selected for histotripsy sessions determines the size and shape of the cavitation zone. Transducers with spherical apertures have ellipsoid focal zones that are 1–2 mm (short axis) by 2–4 mm (long axis). To create a larger treatment area, pulses must be performed repeatedly, and the transducer may be moved mechanically or focused electronically to cover each region being treated. The number of pulses per area varies by organ and can be up to 100 pulses per focal region in organs the liver. After cavitation, the acellular debris is absorbed by the body over ~1–2 months [39].

In addition to tissue composition, tissue-fluid interfaces in the body also affect cavitation and change how target lesions develop. Tissue-fluid interfaces occur within vessels and interstitial spaces and affect bubble formation because the acoustic impedance is different among soft tissues and fluids. The interface can affect how energy transfers from ultrasound and enhance or diminish cavitation depending on the interface properties [29,32,40]. Additionally, tissue hydration affects cavitation, with more hydrated tissues being more favorable for bubble formation and more fibrotic tissues less favorable for cavitation-induced destruction [29]. These interactions are essential to consider when optimizing histotripsy parameters for tissue ablation and minimizing off target destruction.

Histotripsy is unique from other methods which cause tissue destruction. The use of nonthermal mechanical destruction through ultrasound-generated bubbles has several advantages over thermal and surgical methods of tissue destruction. High Intensity Focused Ultrasound (HIFU) is an ultrasound-based method that destroys tissues using long pulses (10–20 ms) and high duty cycles (10–100%). Through this, it causes thermal ablation of tissues by inducing coagulative necrosis, damaging structures within the treatment zone, including vessels [41]. Clinically, HIFU has been used to treat uterine fibroids, neurological disorders, and tumors in the prostate, breast, liver, and pancreas [21]. While direct comparisons between the clinical efficacy of HIFU and histotripsy are difficult due to a lack of robust data, HIFU is known to be limited by long procedure times, restricted treatment locations, and thermal side effects. In contrast, histotripsy uses short pulses and low duty cycles to mechanically fractionate tissues, which reduces thermal side effects and enhances tissue preservation. Microwave ablation is another energy-based technique with similar goals to histotripsy. It uses thermal energy which is delivered through an antenna and causes coagulative necrosis of target tissue. In a healthy porcine model, microwave ablation produced longer and more oblong zones, while histotripsy generated more spherical zones that decreased significantly over time, and it was generally associated with fewer biliary complications [42,43]. Finally, cryoablation also induces tissue destruction through the freezing of tissues and induction of coagulative necrosis which is thought to be slower and less effective than other techniques. Compared to cryoablation, histotripsy had larger ablation zones and faster resorption of treated tissue; it should be noted that this difference in outcome also depends on how many cryoneedles are used in a cryoablation procedure. Histotripsy also had fewer complications and maintained similar functional outcomes in a healthy swine kidney model [42,44,45].

Boiling histotripsy is another method of tissue destruction that uses mechanical properties. Compared to classical histotripsy, it uses lower peak negative pressures and higher shockwaves. The peak negative pressures (10–20 MPa) are lower than histotripsy and higher than HIFU, with a positive pressure shockwave that is typically above 70 MPa [33]. The positive pressures generate boiling bubbles at the focal zone. The boiling bubbles produce rapid micro-jetting and fountain-like projections which mechanically disrupt tissues. The fountain projectiles recirculate and homogenize tissue in the target zone in a process called tissue atomization [34]. The treatment time is typically shorter, between 1 and 30 s in contrast to the range up to several minutes per focal region in traditional histotripsy. Boiling histotripsy is being actively investigated for similar clinical applications as classical histotripsy, including applications in liver and renal cancers [35].

## 3. Histotripsy in Preclinical Cancer Models

**Figure 1:**
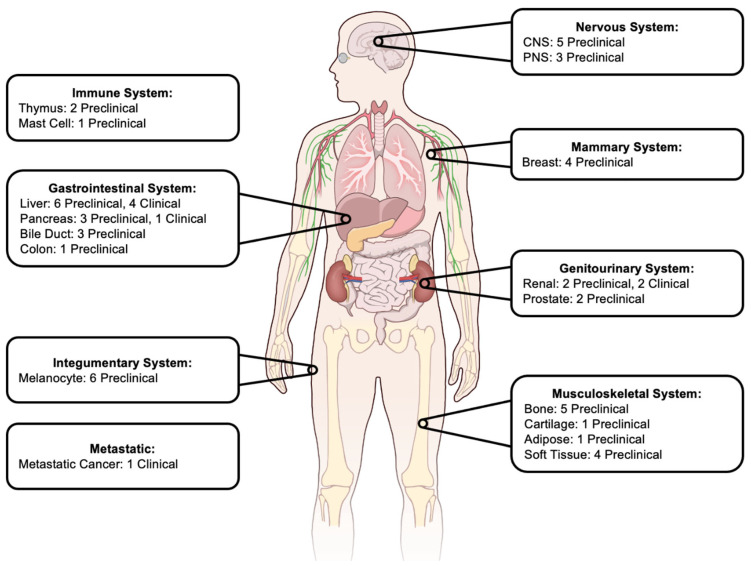
Number of current preclinical and clinical trials using histotripsy. CNS: Central Nervous System; PNS: Peripheral Nervous System. Image Contribution: NIAID Visual and Medical Arts.

Preclinical studies of cancer models enable continued development and refinement of histotripsy technology [46,47,48] (Figure 1). Methodological considerations such as animal selection (small vs. large, species), cancer model (spontaneous tumor development vs. gene editing), and technical settings (frequency, number of elements, aperture size, F-number, FWHM) all influence experimental results [46]. In addition to the wide array of experimental conditions, a large variety of cancer models have been explored ranging from localized tumors to disseminated cancers. The current results of preclinical histotripsy studies are detailed below, organized by location (Table 2).

### 3.1. Nervous System

The nervous system has become a growing focus of histotripsy ablation, focusing on both central nervous tumors [63,64,65,66,67] (gliomas, glioblastomas, meningiomas) and peripheral nervous tumors [68,69,70] (neuroblastoma). For central nervous system tumors, the thickness and density of the skull adds another dimension of complexity due to the bone’s ability to modulate the beam’s accuracy and power [85], similar to focused ultrasound ablation. Despite this additional limitation, histotripsy has demonstrated strong results in orthotopic models, albeit with the caveat of different skull dimensions of murine models [63,64,65,66]. For example, Choi et al. showed improved targeting of glioblastoma using stereotactic targeting [63]. Choi et al. also used histotripsy ablation in glioma with MRI, cross-validated with histology, to show safety profiles [64] (partial repository for MRI available at: https://data.mendeley.com/datasets/c3b563vzzp/1 (accessed on 25 March 2025)). Additionally, glioblastoma therapy using histotripsy showed an elevated immune response with increased IFN-gamma and decreased myeloid-derived suppressor cells [65]. Glioma and associated lung metastasis also showed strong response to histotripsy [66], with a lower number of pulses minimizing hemorrhage while maintaining effective tumor ablation. One study even demonstrated the feasibility of histotripsy in canines when applied to meningiomas [67] while another study used histotripsy to transiently open the blood–brain barrier (BBB), with Duclos et al. showing loss of tight junctions and subsequent repair over a four-week interval [66].

In the peripheral nervous system, neuroblastoma has shown strong results in response to histotripsy [68,69,70]. Boiling histotripsy was able to mechanically fragment subcutaneous neuroblastoma in mice [70]. Histotripsy also induced a stronger immune response [69] and, notably, drove tumor apoptosis [68].

### 3.2. Breast

Preclinical studies of histotripsy applied to breast cancer have demonstrated positive immunological and mechanical results [46,58,59,60,61]. Nam et al. [58] and Hendricks-Wegner et al. [46] showed positive ablation results in 4T1 tumor models, a triple-negative breast cancer model, in both boiling and intrinsic threshold histotripsy, respectively. More recently, Tang et al. [59] applied ultrasound-guided histotripsy to a HER2+ murine model which showed release of HER2 from tumor cells. Additionally, histotripsy demonstrated augmented immune response with increased levels of pro-inflammatory cytokines post-treatment [61,62].

### 3.3. Gastrointestinal

Within the gastrointestinal system, the liver has been the most studied organ [46,49,50,51,52,53] followed by pancreas [46,54,55], bile duct [46,56,57], and colon [58] in preclinical studies. A majority of studies used subcutaneous murine models [46,49,50,53,54,56,57,58] with a more recent transition to orthotopic [51,52,55] and porcine [46,55] models. Hepatocellular carcinoma has demonstrated a strong response to histotripsy with reduction in tumor burden [49,51], improved survival outcomes [52], and potential augmentation when combined with immunotherapy [50,53] (also demonstrated in colon carcinoma [58]). Current work is exploring improved targeting techniques including CT [86] and MRI [87] guided systems. Histotripsy was also effective in subcutaneous pancreatic adenocarcinoma [54] and orthotopic pancreatic ductal epithelial carcinoma [55], with challenges achieving total ablation in the orthotopic model. Cholangiocarcinoma, a tumor with abundant fibrous components, also demonstrated a good response to histotripsy [56,57], with Wegner et al. showing that half of the tumor models treated were undetectable after 2.5 weeks [57].

### 3.4. Genitourinary

Histotripsy has been applied to both prostate [81,82] and renal cancer [83,84]. For the prostate, Schade et al. utilized a transabdominal approach in canines which showed homogenization of the tumor [81] while Chevillet et al. found complete liquefaction of a subcutaneous prostate model in mice with increased tumor-derived microRNA post-treatment [82]. For the kidney, Styn et al. utilized histotripsy to target a VX-2 tumor model in a rabbit kidney [83]. Their study found extensive inflammatory reaction and fractionation of malignant tissue on histology [83]. Similarly and more recently, Schade et al. used boiling histotripsy on de novo renal cell carcinoma in Eker rats [84]. Alongside the focal intra-parenchymal damage with clear borders, plasma showed increased levels of TNF along with transient increases in HMGB1, IL-10, and IL-6 [84]. Most notably, both the treated and untreated contralateral kidney showed increased CD8+ T cell infiltration at 48 h post-treatment, demonstrating a global increase in immune response [84].

### 3.5. Musculoskeletal

In contrast to other organ systems, histotripsy studies involving the musculoskeletal system have predominantly used spontaneous tumors in large animal models including canines [46,60,75,76,77,79,80] and felines [78] instead of specific murine models. Histotripsy has been applied to a variety of musculoskeletal tumors including fibrous osteosarcoma [46,75,77,79,80], chondrosarcoma [75,77], soft tissue sarcoma [46,60,76,78], and lipoma [60] with mixed results. Histotripsy showed promising results in osteosarcoma with safe and effective margins in numerous studies [46,75,77,79,80] across a variety of bones (femur, tibia, radius) and subtypes (osteoblastic, telangiectatic, chondroblastic). Similarly, Ruger et al. found positive results in both canines [76] and felines [78] with soft tissue sarcoma. In the feline group, target regions showed ablative damage with an increase in IBA-1 positive cells with no associated cytokine change [78]. Conversely, Ashar et al. found progressive disease within 60 days after treatment in both low-grade soft tissue sarcoma and lipoma [60].

### 3.6. Integumentary

One major focus of preclinical studies of histotripsy is on treating melanoma [50,53,70,71,72,73]. For example, Hoogenboom et al. demonstrated fragmentation of a soft tissue melanoma in both a 100 and 200 pulses per focal point study, with improved results in the 200-pulse arm [70]. However, in addition to studying the mechanical results of histotripsy in melanoma, there is a growing interest in its immunological consequences. One study found that histotripsy combined with in situ administration of αCD40 (HT + αCD40: HT40) improved response to immune checkpoint inhibitors [71]. Three additional studies demonstrated abscopal immune response and improved immunological environments in response to histotripsy treatment [50,53,72]. Notably, one recent study by Song et al. linked histotripsy and hypoxia abrogation [73], demonstrating that histotripsy can lead to rapid loss of intratumoral hypoxia, suppression of HIF-1α, and a decrease in downstream pro-survival proteins followed by an upregulation of CXCR3 ligand CXCL10 and CXCR3+/CD8+ T cell infiltration. Song et al. also demonstrated improved effects of histotripsy on melanoma models when combined with Trametinib, a selective mitogen-activated extracellular signal-regulated kinase (MEK) inhibitor [73].

### 3.7. Immune System

Histotripsy applied to immunological cancers has been limited in scope with mixed results. MR-guided boiling histotripsy demonstrated fragmentation of a subcutaneous thymoma murine model with sharp treatment borders [70,74]. Meanwhile, spontaneous mast cell tumors in canines showed progressive disease despite treatment [60].

## 4. Clinical Trials

The THERESA study, conducted in Barcelona in 2019, is the first histotripsy feasibility trial. It included 8 patients with 11 unresectable multifocal liver tumors [88]. Investigators defined the primary endpoint as tissue destruction matching the target volume, which was assessed by a post-procedure MRI. This goal was achieved in all procedures, with no significant adverse effects. Primary outcome measures typically focused on technical success, which was determined by the treatment volume being greater than or equal to the targeted tumor. Adverse effects could include any deleterious effects during or after the procedure. This includes but is not limited to bleeding, infection, or unintended damage to surrounding tissues.

HistoSonics Inc., a medical device company that used the science of histotripsy to develop the Edison device, began a multi-center, single-arm non-randomized prospective trial in 2021 to investigate histotripsy’s safety and efficacy in liver tumors in Europe [89]. HistoSonics named this trial #HOPE4LIVEREU/UK and began an identical trial entitled #HOPE4LIVERUS in the US; the data from these trials were then pooled to submit to the FDA. The principal investigators of the trials were from the University of Michigan and University of Wisconsin. In September 2023, HistoSonics presented data from both trials at the Cardiovascular and Interventional Radiological Society of Europe meeting [90]. At this point, 42 out of 44 tumors had been successfully treated in 40 subjects, yielding an efficacy rate of 95.5%. The two tumors that were not successfully treated were noted to be due to user, not device error, since both tumors were mistargeted. The investigators also encountered three complications due to known risks of focal liver therapies, and not complications specific to histotripsy. Two participants had grade 3 events as per the Common Terminology Criteria for Adverse events (sepsis in someone with a biliary stent and pleuritic pain that required admission) and one had a grade 5 event (hepatic failure 12 days post-procedure in a patient with significant breast primary metastatic lesions which eventually led to death 37 days after the procedure) [24]. Ultimately, the 7% rate of major post-procedure complications was quite reassuring. Moreover, a comprehensive safety analysis of 230 cases of histotripsy procedures targeting liver tumors found that only 12 experienced any complications; among these, 3 severe cases were due to malignancy progression and had histotripsy specifically performed for palliative care [25]. Overall, these findings underscore histotripsy’s exceptional safety profile.

Including the #HOPE4LIVER trials, eight clinical trials (including those withdrawn or terminated) are examining histotripsy applications in oncology: 4 in liver tumors, 2 in renal tumors, 1 in pancreatic adenocarcinoma, and 1 in metastatic disease [91]. Table 3 provides a comprehensive overview of each trial. These investigations emphasize safety parameters, technical efficacy, and overall clinical viability. Notably, these protocols have rigorous inclusion and exclusion criteria when it comes to patient age, comorbidities, tumor dimensions, and organ function. Protocols are also beginning to investigate combination approaches, like histotripsy alongside immune checkpoint inhibitors—a direction that will likely be of major clinical interest moving forward. Trial sizes range from smaller cohorts of 20–50 participants to large investigations with thousands of subjects. The research is being conducted globally by multiple sponsors ranging from academic institutions to industry.

Histotripsy continues to undergo thorough assessment across cancer types, standing at the vanguard of novel clinical tools in oncology. Currently, the most exciting future uses beyond liver tumors are renal and pancreatic cancers. Histotripsy’s mechanism of destruction could preserve the function of the kidney’s urine-collecting system, which is something that more invasive interventions have difficulty achieving. Similarly, applying it to patients with unresectable pancreatic tumors could expand treatment options for individuals who previously had none. Continued investigation will be necessary to establish its efficacy and safety across various populations.

## 5. Histotripsy in Current Applications

Currently, histotripsy is being explored in broad preclinical applications ranging from aortic stenosis and benign prostatic hyperplasia to the treatment of solid tumors [21,92,93]. However, as of late 2023, histotripsy has received FDA market authorization for the non-research manufacture and clinical use of the Edison platform (HistoSonics) to treat primary and metastatic liver tumors [94]. This approval was made soon after the success of the US arm of the #HOPE4LIVER phase I/II clinical trial for liver tumor treatment [24]. A recent multicenter safety profile analysis of histotripsy in liver tumor treatment found only 1% of patients experienced major complications 30 days after treatment, and further, within patients with complications, all major complications were attributed to disease progression rather than treatment-related morbidity [25]. Also, as mentioned previously, early-stage clinical trials are underway for renal carcinoma and pancreatic adenocarcinoma treatment as of 2025.

Histotripsy’s ability to spare large vessels like the hepatic portal vein and pancreatic duct makes it a promising technology for use in delicate abdominal areas that are untreatable by current invasive surgical techniques [24]. One study outlined T1-weighted MRI of the liver after treatment of a primary liver tumor near the hepatic portal vein in a porcine model and reported negligible damage to the vessel [20]. Where surgery, radiation, and methods like HIFU and other thermal ablative modalities cannot be utilized, histotripsy offers a promising, patient-saving avenue due to its capability to precisely and locally destroy tissue in low-collagen, bulk tissue environments, like those of solid tumors in the liver, kidney, and pancreas. From a clinical standpoint, histotripsy is considered a focal therapy similar to microwave ablation and other ablation techniques, but it offers additional advantages, including tissue specificity, non-thermal ablation, and a non-invasive approach. Specific guideline recommendations for selecting between histotripsy and conventional cancer therapies are still under development and remain largely institution- and clinician-dependent. Comprehensive decision-making frameworks require further technical validation and consideration of patient-specific factors and are therefore beyond the scope of this review. Nonetheless, in cases involving hepatic lesions adjacent to delicate, collagen-rich structures, or in patients with comorbidities that contraindicate traditional thermal ablation, histotripsy may currently offer a preferable therapeutic alternative.

### 5.1. The Ideal Candidate

**Figure 2:**
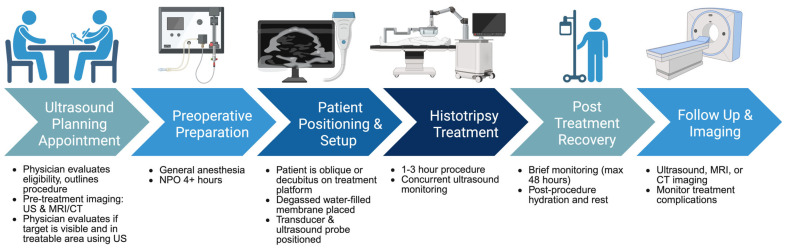
Overview of current clinical workflow of histotripsy procedures.

Figure 2 visualizes the standard patient histotripsy journey and typical patient demographics, from initial work-up and decision making to post-treatment clinical follow-up. The implementation of histotripsy in current patient treatment strategies depends on certain patient demographics and requirements. To determine which specific patients are suitable candidates for histotripsy, it is necessary to consider the pathological condition to be treated. In the solid tumor space, the liver is currently the only organ with standard-of-care clinical protocols that authorize histotripsy usage. However, patient selection criteria can also be posited for kidney and pancreatic malignancies since both cancer types are undergoing clinical trials for histotripsy treatment as of early 2025.

For the liver in particular, an ideal patient for histotripsy treatment must have hepatic tumors each of less than approximately 4 cm in diameter, but multiple lesions within the liver can be treated in a single procedure; tumors should also be in favorable locations, including within the 14 cm histotripsy depth-of-penetration limit and not proximal to collagen-poor major vessels or other organs susceptible to ultrasonic damage [95,96,97]. Certain centers further limit the number and size of primary tumors outside of the liver when treating hepatic metastases [95]. For patients who have previously received a Whipple procedure, or pancreaticoduodenectomy, histotripsy may still be possible, but patients may require prolonged antibiotics to minimize infection risk or abscess formation [97]. As histotripsy becomes more ingrained in liver cancer treatment across the United States, standardization of clinical protocols will surely develop through guidelines by governing bodies like the Society of Interventional Radiology or National Comprehensive Cancer Network.

For clinical trials involving the liver, patient eligibility varies from standard of care guidelines and between clinical trials. Please refer to Section 4 and Table 3 for more information on patient inclusion and exclusion criteria. Pancreas and kidney tumor histotripsy treatments are also currently under clinical trial investigation at phases I and III, respectively, and their patient selection criteria are also listed in Section 4.

### 5.2. The Typical Procedure

The typical procedure for a histotripsy patient begins with consultation and surgical planning with an interventional radiologist or other proceduralist specialized in usage of the technology [98]. During these initial visits, proceduralists will evaluate the patient for potential effectiveness of treatment, and outline the procedure and area to be treated, along with potential risks and side effects of histotripsy. Pre-imaging is factored into patient eligibility for treatment. This imaging includes both ultrasound, used to examine the liver to ensure treatment efficacy, and MRI/CT, to locate and measure the tumor [98]. Pre-procedural planning involves determining the treatment area and size, planning orientation of the histotripsy system, and patient body positioning.

Currently, patients are treated under general anesthesia and should be NPO prior to the procedure per local hospital guidelines, due to potential complications with sedation and liver function postprandially [95,98]. General anesthesia is used to ensure the patient remains still during the intraoperative planning and treatment portions. Jet ventilation (low-volume, high-frequency), dual lumen intubation, and single lung ventilation are occasionally used to help minimize respiratory movement and further improve treatment accuracy [99].

While anesthetized the patient is positioned on a platform in a supine, oblique, or decubitus position. A flexible acoustically transparent membrane is positioned above the liver area and filled with degassed water to serve as a coupling medium for the device transducer [98]. This water is maintained at a narrow temperature range of 37 ± 4 °C. Degassing the water and maintaining this temperature ensure that the ultrasound signal transmission is consistent and bubble clouds can be effectively controlled over a medium with consistent acoustic properties [100]. A treatment head, complete with a 1 MHz histotripsy transducer and integrated coaxially aligned ultrasound probe is then positioned over the water-filled membrane and liver area [56]. A robotic arm with 6 DOF motion is utilized to move the treatment head into the water bath. The embedded diagnostic ultrasound probe in the treatment head is manipulated until the prescribed treatment zone encompasses the target with an appropriate margin. Once the treatment zone is selected, it is verified from several obliquities and the voltage required for cavitation is tested at several points within the treatment area. The treatment then proceeds autonomously as the device controls the movement of the robotic arm.

The typical histotripsy procedure lasts about 1.5 to 3 h from preoperative preparation and planning to postoperative monitoring, with actual treatment times taking roughly 10–40 min. The treatment head, operated by a trained physician and precisely registered computer homing program, delivers focused ultrasound bubble clouds to the targeted malignant areas in 3 to 6 mm incremental movements; these bubble clouds simultaneously provide precise tumor destruction and contrast for ultrasound confirmation that cavitation is occurring [98,99,101]. No incisions or needles are utilized during the procedure.

The indication for multiple procedures and the decision to operate on multiple tumors in one procedure largely depend on tumor number, size, and location metrics. Overall patient health is also factored into clinician decision making. In general, patients undergo treatment for all liver tumors in one setting, as the actual procedure time is relatively short compared to manual surgical resection techniques, and the safety profile for multiple-tumor treatment in a single session is strong [88]. The THERESA trial further indicated that, for patients requiring multiple procedures, up to three procedures were allowed per patient, and a mandatory monthlong interval between procedures had to be adhered to [88]. Interestingly, initial reports on the treatment of pancreatic cancer demonstrate a potential beneficial impact on survival rates when patients are treated in multiple sessions [102].

Most patients recover within hours and do not require overnight hospitalization; however, depending on factors like complexity of procedure, pain management, and support availability at home, patients may spend 24 to 48 h in the hospital for monitoring [103]. Patients are advised to stay hydrated and rest immediately post-procedure, with some patients experiencing discomfort or slight pain at the treatment site in the following days of recovery. Follow-up imaging is utilized to monitor treatment effects and potential complications, as well as to serve as a baseline for comparison in future follow-up scans.

### 5.3. Peri- and Post-Operative Imaging

Histotripsy treatment is currently clinically monitored using standard diagnostic B-mode ultrasound. Nonetheless, other ultrasound-based techniques and imaging modalities have also been explored in the evaluation of histotripsy-related factors like bubble-cloud localization, tissue elasticity, image guidance, and treatment efficacy, albeit mostly in the preclinical setting.

Ultrasound plays the predominant role in histotripsy procedure monitoring, used primarily for bubble cloud visualization, multiplanar imaging, and color Doppler imaging [101]. However, within ultrasound, there are several research sub-modalities applied preclinically for the study of various histotripsy-related effects. A 2019 study examined plane-wave B-mode ultrasound imaging in the observation of histotripsy bubble clouds, demonstrating the potential for bubble clouds to remain unshifted over the course of a pulse tissue excitation [104]. Another study examining shear-wave elastography- applied in an ex vivo porcine kidney model to visualize the tissue elasticity change in response to bubble cloud formation and tissue destruction- provides promise in the precise characterization of treatment-induced lesions, which remains a current issue with standard-of-care B-mode ultrasound in the immediate post-treatment imaging setting [105]. Other studies also improve imaging contrast over standard plane-wave imaging by utilizing chirp-coded excitation and signal post-processing techniques like subharmonic and Volterra filtering, establishing abundant and alternative methods for bubble cloud localization in different histotripsy use cases [106].

Magnetic resonance (MR) guided approaches have been employed for applications ranging from singular bubble cloud detection in intact porcine liver and brain tissue to R2 and Apparent Diffusion Coefficient contrast parameter response to histotripsy ablation levels in porcine liver, kidney, and muscle tissue [107,108]. In one study, T2-weighted transcranial MR was explored in conjunction with bioluminescence imaging and concurrent histology to assess the effects of murine glioma ablation hours after treatment [66]. A recent study further explored MRI-guided histotripsy in an ex vivo bovine brain within a human skull; MR, in conjunction with acoustic radiation force imaging (ARFI) and thermometry, was shown to accurately perform pre-treatment histotripsy targeting, demonstrating the feasibility to implement high-resolution imaging through bone during the localization process prior to ablation [109]. MR approaches remain largely experimental but provide promise for future iterations of the histotripsy operative environment. T1 and T2-weighted imaging are further useful in post-treatment follow-up imaging, as treated areas are hyperintense in appearance [101,110].

X-ray technology, specifically through standard and cone-beam computed tomography (CT), also provides excellent and rapid imaging, which may expand our ability to localize and treat tumors invisible to standard ultrasound, or deep tumors and tumors which are not easily seen with ultrasound due to dense overlying structures, as is outlined in a group of studies [111,112,113]. Post-treatment long-term imaging is often performed with either MR or CT [101].

Ultimately, while ultrasound, contrast-enhanced CT, and MRI are the most used post-treatment imaging options, recent work has also explored new modalities for assessing treatment response after histotripsy [101]. Contrast-enhanced ultrasound (CEUS) is an effective, fast, and well-tolerated technique with high resolution in tissue perfusion and lesion characterization [114]. It is useful for visualizing vessels and detecting residual disease; in a recent hepatic tumor study, it was effectively used to monitor post-treatment perfusion and lesion extent [114]. While other imaging methods are being investigated for assessing histotripsy efficacy in ex vivo and in vitro tissues, they have yet to be validated in clinical studies [115]. Post-treatment long-term imaging is most often performed with either MR or CT [101].

### 5.4. Monitoring and Follow-Up

As stated previously, post-operative monitoring for patients after histotripsy involves a combined paradigm of imaging, symptom assessment, and side effect management, in combination with adjacent therapies for the patients’ underlying malignancy. Imaging entails short-term observation of the ablated site using ultrasound, and long-term characterization of tissue changes at regular intervals in the weeks and months following tissue destruction, traditionally using contrast-enhanced T1- or T2-weighted MRI, CT, or ultrasound. Imaging primarily focuses on the treated area and surrounding organs and vascular structures to ensure there is no unintended collateral damage. In experimental study of the abscopal effect of histotripsy, distal organs and structures may also occasionally be imaged for the purpose of examining the intended systemic effect [116].

Patients may attend post-operative clinical visits to assess how the treatment was tolerated acutely, usually within 1–2 weeks post-procedure, and they may also be seen at regular monthly intervals. Short term visits are intended for review of initial imaging results, assessment of acute symptoms like pain and discomfort at the tissue site, and dietary and lifestyle advice [103]. Follow-up long-term visits include physical examination and further imaging review, as well as standard monitoring for signs of disease recurrence or long-term complications. Based on long-term histotripsy tissue response, concurrent therapies can also be adjusted at follow-up appointments.

Symptoms range from acute to chronic after histotripsy treatment; however, most patients experience minimal symptoms due to the non-invasive nature of the technology. It is not uncommon for patients to have mild discomfort at the site of treatment, and there are rare reports of severe pain [21,103]. Chronic effects have not been adequately captured yet due to the relative novelty of the technology; however, experimentally, they can include scarring at the ablation site in the liver, though these observations are minimal [52,56].

Overall, histotripsy offers a highly favorable recovery profile compared to traditional ablation strategies. Its non-invasive nature minimizes acute and chronic symptoms while allowing rapid return to normal activities. Post-operative monitoring through advanced imaging modalities ensures precise assessment of treatment success and long-term outcomes.

## 6. Limitations and Future Work

Histotripsy offers a relatively low risk profile due to its targeted therapeutic effect and noninvasive nature; nonetheless, some complications have been reported in the literature. Complications during procedures primarily involve vascular thrombosis through cavitation-induced platelet activation and aggregation [43]. Theoretical risks of tumor lysis and metastasis due to the disruptive effect of cavitation are unfounded in studies examining the systemic effects of histotripsy [50,117]. Late complications following treatment largely revolve around discomfort at the treatment site, with occasional reports of minimal tissue scarring, and the potential for infection, hematoma, pleuritis, or other effects due to unintended disruption of tissue near the liver treatment area [101]. Also, theoretically, there is a risk for rupture of smaller collagen-lacking vasculature, though this has not been reported clinically so far [35]. Overall, the 30-day risk of complication following liver histotripsy treatment stands at 7%, much less than other competing treatment methods [24]. Promisingly, in a recent post-trial safety profile of histotripsy across 18 centers and nearly 300 patients, only 12 complications were reported according to the Clavien–Dindo classification and Comprehensive Complication Index, and all were graded as either minor or attributed to underlying disease progression [25]. Though rare, one patient in the #HOPE4LIVER trial developed liver failure following treatment and subsequently died. Strategies to mitigate these risks include clear delineation of surgical margins and operation only in areas uncomplicated by surrounding biological structures, though no device-related deaths have been reported since histotripsy was introduced into clinical implementation.

Histotripsy shows promise in liver tumor treatment and is undergoing clinical evaluation for other organs; however, there are limitations to its clinical use. Organs containing gas, like the lungs and gastrointestinal system, are not suitable for histotripsy due to the high acoustic impedance mismatch for ultrasound and the potential for massive collateral damage within these structures [21]. Additionally, bone-shielded organs like the brain, while currently being explored in preclinical studies, still pose a challenge for treatment due to the attenuation of signal through dense mediums and the potential for skull-mediated ultrasound beam distortion. For a similar reason, certain areas of the pancreas may be more difficult to treat, as they lie deep in the retroperitoneum and could experience acoustic shadowing. More generally, careful selection of acoustic windows is necessary in all tissues for effective delivery of ultrasonic therapy. As histotripsy parameters are further tuned and standardized, these challenges will likely be addressed. Other limitations of the treatment include protracted acellular tissue debris clearance times and a limited understanding of long-term outcomes due to the relative novelty of the technology in a widely adopted clinical space [101].

Histotripsy has potential for clinical application in many other cancers and pathologies in the future, based on the diversity of currently detailed animal studies and clinical trials. With the potential to be used as a conditioning regimen for patients undergoing liver transplantation, histotripsy provides more than mere standard malignancy treatment [118]. Of additional interest are histotripsy’s future roles in immunomodulation and combination with immunotherapy [119]. Histotripsy is preliminarily being explored for its immune-boosting properties, from its ability to shift the local tumor environment from immunologically cold to hot, to its part in enhancing both the innate and adaptive immune anti-tumor responses systemically [22,120,121,122]. Histotripsy has been shown to release a rich mixture of damage-associated molecular patterns (DAMPs), specifically HMGB1, extracellular/extranuclear DNA, and ATP [54,123], which can activate intracellular and extracellular receptors resulting in pro-inflammatory cytokine production. Pepple et al. and Qu et al. have described the dual local and abscopal inhibitory effect of histotripsy on tumor growth; locally treated tumors primed innate immune activation of myeloid and natural killer cells in the short term for further damage, while anti-tumor CD8+ T cells acted in a delayed manner to maintain the local environment and target off-site untreated metastases [50,53]. These studies, among others, further demonstrated the potent synergistic value of histotripsy in combination with checkpoint inhibitor therapies like anti-PD-L1, anti-CTLA-4, and agonistic CD40 agents [50,52,53,71]. This synergistic effect is supported by both histotripsy and checkpoint inhibitors being shown to activate ferroptosis, iron-dependent programmed cell death [53,124]. Because histotripsy non-thermogenically destroys tumor tissue and releases intact immunologically recognizable antigens in its wake, these delayed beneficial systemic responses are possible, in stark contrast to thermal (HIFU) or ionizing therapies like radiation which wholly destroy similar antigens [22]. Histotripsy is likely to impact the entire treatment landscape for solid malignancy, beyond its immediate obvious impact in non-invasive mechanical tissue destruction.

## 7. Conclusions

Histotripsy is a transformative, non-invasive modality that ultimately addresses many of the key limitations of current ablative and surgical techniques in oncology and beyond. Its high level of precision, favorable safety profile, and potential systemic benefits are making it possible for patients who might have previously gone untreated to obtain potentially life-saving care. Preclinical studies with a variety of animals, models, and settings have highlighted histotripsy’s potential in everything from central nervous system, to genitourinary, to immunological cancers. Clinical trials in liver tumors have also demonstrated high efficacy rates and low complication rates. Ongoing studies in liver, as well as renal and pancreatic malignancies underscore the potential for this technology to revolutionize oncological treatment across several domains. While certain limitations must still be addressed, including challenges in areas of the body covered by bone or filled with gas, results are promising. Histotripsy is well-positioned to become a mainstay in cancer care.

## Figures and Tables

**Table 1:** Summary of key acoustic parameters across ultrasound cavitation modalities, including intrinsic threshold histotripsy, shock-scattering histotripsy, HIFU, and boiling histotripsy. Data reported from references [26,29,30,31,32,33,34,35].

Ultrasound Parameters				
Parameter	Intrinsic Threshold Histotripsy	Shock-Scattering Histotripsy	HIFU	Boiling Histotripsy
Frequency	250 kHz–3 MHz	500 kHz–3 MHz	1–5 MHz	1–5 MHz
Peak Negative Pressure	26–30 MPa	15–20 MPa	5–10 MPa	10–20 MPa
Peak Positive Pressure	N/A	>50 MPa	<30 MPa	>70 MPa
Duty Cycle	<1%	<1%	10–100%	<2%
Pulse Duration	1–2 cycles	3–10 cycles	10–20 ms	High duty cycle
Pulse Repetition Frequency	1 Hz–1 kHz	1 Hz–1 kHz	–	1 Hz–2 kHz
Mechanism	Mechanical cavitation	Shock-scattering cavitation	Thermal coagulative necrosis	Boiling mechanical disruption

**Table 2:** Preclinical trials using histotripsy.

Study	Organ System	Cancer Model
Histotripsy for Non-Invasive Ablation of Hepatocellular Carcinoma (HCC) Tumor in a Subcutaneous Xenograft Murine Model (Worlikar et al., 2018) [49]	Liver: Hepatocellular Carcinoma	Subcutaneous Hep3B tumors in NSG and NOD-SCID mice
Non-thermal histotripsy tumor ablation promotes abscopal immune responses that enhance cancer immunotherapy (Qu et al., 2020) [50]	Liver: Hepatocellular Carcinoma Integumentary: Melanoma	Subcutaneous Hepa1-6 hepatocellular carcinoma in C57BL/6 mice Subcutaneous B16GP33 melanoma in C57BL/6 mice
Effects of Histotripsy on Local Tumor Progression in an in vivo Orthotopic Rodent Liver Tumor Model (Worlikar et al., 2020) [51]	Liver: Hepatocellular Carcinoma	Orthotopic N1-S1 hepatic tumors in immunocompetent Sprague Dawley rats
Impact of Histotripsy on Development of Intrahepatic Metastases in a Rodent Liver Tumor Model (Worlikar et al., 2022) [52]	Liver: Hepatocellular Carcinoma	Orthotopic McA-RH7777 hepatic tumors in immunocompetent Sprague Dawley rats
Spatiotemporal local and abscopal cell death and immune responses to histotripsy focused ultrasound tumor ablation (Pepple et al., 2023) [53]	Liver: Hepatocellular Carcinoma Integumentary: Melanoma	Subcutaneous Hepa1-6 hepatocellular carcinoma in C57BL/6 mice Subcutaneous B16F10 melanoma in C57BL/6 mice
Histotripsy Ablation in Preclinical Animal Models of Cancer and Spontaneous Tumors in Veterinary Patients: A Review (Hendricks-Wegner et al., 2021) [46]	Bile Duct: Cholangiocarcinoma Breast: Stage IV Breast Cancer Renal: Renal Cell Carcinoma Pancreas: Epithelioid Carcinoma Liver: Hepatocellular Carcinoma Soft Tissue: Sarcoma Bone: Osteosarcoma	Subcutaneous patient-derived xenograft cholangiocarcinoma in immunocompromised NSG mice Orthotopic 4T1 mammary tumor in BALB/c mice Subcutaneous Renca RCC in BALB/c mice Subcutaneous Panc01 in RAG2/IL2RG deficient pigs Subcutaneous HepG2 in RAG2/IL2RG deficient pigs Spontaneous soft tissue sarcoma in canines Spontaneous hindlimb osteosarcoma in canines
Histotripsy Ablation Alters the Tumor Microenvironment and Promotes Immune System Activation in a Subcutaneous Model of Pancreatic Cancer (Hendricks-Wenger et al., 2021) [54]	Pancreas: Pancreatic Adenocarcinoma	Subcutaneous Pan02 in C57/Bl6 mice
Successful In Situ Targeting of Pancreatic Tumors in a Novel Orthotopic Porcine Model Using Histotripsy (Imran et al., 2023) [55]	Pancreas: Ductal Epithelial Carcinoma	Orthotopic Panc-1 in RAG2/IL2RG double-knockout pigs
Histotripsy for the Treatment of Cholangiocarcinoma Liver Tumors: In Vivo Feasibility and Ex Vivo Dosimetry Study (Hendricks-Wenger et al., 2021) [56]	Bile Duct: Cholangiocarcinoma	Subcutaneous patient-derived xenograft cholangiocarcinoma in immunocompromised NSG mice
Histotripsy for the Treatment of Cholangiocarcinoma in a Patient-Derived Xenograft Mouse Model (Hendricks-Wenger et al., 2022) [57]	Bile Duct: Cholangiocarcinoma (Adenosquamous Carcinoma)	Subcutaneous patient-derived xenograft of adenosquamous carcinoma (TM01225), a subtype of cholangiocarcinoma, in immunocompromised NSG mice
Investigation of the Potential Immunological Effects of Boiling Histotripsy for Cancer Treatment (Nam et al., 2020) [58]	Colon: Colon Carcinoma Breast: Triple-negative Breast Carcinoma	Subcutaneous CT26 colorectal in BALB/c mice Subcutaneous 4T1 triple-negative breast carcinoma in BALB/c mice
Ultrasound-Guided Histotripsy Triggers the Release of Tumor-Associated Antigens from Breast Cancers (Tang et al., 2025) [59]	Breast: HER-2+ Breast Cancer	Spontaneous HER2-overexpressing E0771E2 in C57BL/6 HER2 transgenic mice
Enabling Chemo-Immunotherapy with HIFU in Canine Cancer Patients (Ashar et al., 2024) [60]	Breast: Mammary Mass Breast: Papillary Adenocarcinoma Musculoskeletal: Soft Tissue Sarcoma (low-grade) Musculoskeleta: Lipoma Immune System: Mass Cell Tumor (low-grade)	Spontaneous tumor in canines
Boiling Histotripsy Using Dual-Frequency Protocol on Murine Breast Tumor Model and Promotes Immune Activation (Qi et al., 2023) [61]	Breast: Triple-negative Breast Carcinoma	Subcutaneous 4T1 in BALB/c mice
Histoplasty Modification of the Tumor Microenvironment in a Murine Preclinical Model of Breast Cancer (Pieper et al., 2024) [62]	Breast: Triple-negative Breast Carcinoma	Subcutaneous 4T1 in BALB/c mice
Stereotactic Transcranial Focused Ultrasound Targeting System for Murine Brain Models (Choi et al., 2020) [63]	Central Nervous System: Glioblastoma	Orthotopic GL261-luciferase in B6 albino mice
Histotripsy Treatment of Murine Brain and Glioma: Temporal Profile of Magnetic Resonance Imaging and Histological Characteristics Post-treatment (Choi et al., 2023) [64]	Central Nervous System: Glioma	Orthotopic GL261 in BL6 mice
Histotripsy Mediated Immunomodulation in a Mouse GL261 Intracranial Glioma Model (Gerhardson et al., 2018) [65]	Central Nervous System: Glioblastoma	Orthotopic GL261-luc2 in C57 BL/6 albino mice
Transcranial histotripsy parameter study in primary and metastatic murine brain tumor models (Duclos et al., 2023) [66]	Central Nervous System: Glioma Central Nervous System: Lung Metastasis	Orthotopic GL261 in C57BL/6 mice Orthotopic LL/2-Luc2 in C57BL/6 mice
First-In-DOg HISTotripsy for Intracranial Tumors Trial: The FIDOHIST Study (Vezza et al., 2024) [67]	Central Nervous System: Meningiomas	Spontaneous tumor in canines
Histotripsy induces apoptosis and reduces hypoxia in a neuroblastoma xenograft model (Iwanicki et al., 2023) [68]	Peripheral Nervous System: Neuroblastoma	Orthotopic NGP-luciferase in NCR nude mice
High-Intensity Focused Ultrasound (HIFU) Triggers Immune Sensitization of Refractory Murine Neuroblastoma to Checkpoint Inhibitor Therapy (Eranki et al., 2020) [69]	Peripheral Nervous System: Neuroblastoma	Subcutaneous Neuro2a in A/J mice
Impact of MR-guided boiling histotripsy in distinct murine tumor models (Hoogenboom et al., 2017) [70]	Peripheral Nervous System: Neuroblastoma Integumentary: Melanoma Immune System: Lymphoma	Subcutaneous 9464D in C57Bl/6NCrl mice Subcutaneous B16OVA in C57Bl/6NCrl mice Subcutaneous EL4 in C57Bl/6NCrl mice
Boiling histotripsy and in situ CD40 stimulation improve the checkpoint blockade therapy of poorly immunogenic tumors (Singh et al., 2021) [71]	Integumentary: Melanoma	Subcutaneous B16F10 in C57BL/6 mice
Focused ultrasound ablation of melanoma with boiling histotripsy yields abscopal tumor control and antigen-dependent dendritic cell activation (Thim et al., 2024) [72]	Integumentary: Melanoma	Subcutaneous B16F10-ZsGreen in C57Bl/6J mice
Histotripsy-Focused Ultrasound Treatment Abrogates Tumor Hypoxia Responses and Stimulates Antitumor Immune Responses in Melanoma (Song et al., 2025) [73]	Integumentary: Melanoma	Subctuaneous B16F10 or YUMM1.7 in immunocompetent or CD8-deficient C57BL/6 mice
In vivo MR guided boiling histotripsy in a mouse tumor model evaluated by MRI and histopathology (Hoogenboom et al., 2016) [74]	Immune System: Thymoma	Subcutaneous EL4 in C57Bl/6NCrl mice
Characterizing the Ablative Effects of Histotripsy for Osteosarcoma: In Vivo Study in Dogs (Ruger et al., 2023) [75]	Musculoskeletal: Bone	Spontaneously arising osteosarcoma and chondrosarcoma in canines
Mechanical High-Intensity Focused Ultrasound (Histotripsy) in Dogs with Spontaneously Occurring Soft Tissue Sarcomas (Ruger et al., 2023) [76]	Musculoskeletal: Soft Tissue	Spontaneously arising soft tissue sarcoma in canines
Histotripsy Ablation of Spontaneously Occurring Canine Bone Tumors (Ruger at al., 2022) [77]	Musculoskeletal: Bone	Spontaneously arising osteosarcoma and chondrosarcoma in canines
Histotripsy ablation for the treatment of feline injection site sarcomas: a first-in-cat in vivo feasibility study (Ruger et al., 2023) [78]	Musculoskeletal: Soft Tissue	Spontaneously arising soft tissue sarcoma in felines
Histotripsy Ablation of Bone Tumors: Feasibility Study in Excised Canine Osteosarcoma Tumors (Arnold et al., 2021) [79]	Musculoskeletal: Bone	Spontaneous osteosarcoma tumors in canines in 7.5% gelatin in degassed saline tissue phantom
Investigating cell death responses associated with histotripsy ablation of canine osteosarcoma (Hay et al., 2023) [80]	Musculoskeletal: Bone	Spontaneously arising osteosarcoma in canine patients
Histotripsy focal ablation of implanted prostate tumor in an ACE-1 canine cancer model (Schade et al., 2012) [81]	Prostate: Prostate Cancer	Orthotopic ACE-1 prostate tumor in canines
Release of Cell-free MicroRNA Tumor Biomarkers into the Blood Circulation with Pulsed Focused Ultrasound: A Noninvasive, Anatomically Localized, Molecular Liquid Biopsy (Chevillet et al., 2016) [82]	Prostate: Prostate Cancer	Subcutaneous MatLyLu cells in Copenhagen rats
Histotripsy of VX-2 tumor implanted in a renal rabbit model (Styn et al., 2010) [83]	Kidney: Anaplastic Squamous Cell Carcinoma	Orthotopic VX-2 tumor implanted in New Zealand rabbits
Boiling Histotripsy Ablation of Renal Cell Carcinoma in the Eker Rat Promotes a Systemic Inflammatory Response (Schade et al. 2019) [84]	Kidney: Renal Cell Carcinoma	Spontaneous renal cell carcinoma in Eker rat model

**Table 3:** Clinical trials using histotripsy.

Name of Study	Status	Estimated Completion	Sponsor	Clinical Trials ID	Cancer Type	Enrollment or Estimated	Inclusion Criteria	Exclusion Criteria	Primary Outcome Measures	Secondary Outcome Measures (if Included)
Histotripsy (HistoSonics^®^) for Liver Tumours	Not yet recruiting	2028-09-01	The University of Hong Kong	NCT06579833	primary or secondary liver tumors	20	Fit for general anesthesia Liver tumor size < 10 cm Solitary or multifocal Primary liver tumor such as hepatocellular carcinoma or intrahepatic cholangiocarcinoma Secondary liver tumor such as liver metastasis Patients with operable or inoperable liver tumors Liver transplant candidates awaiting for liver graft	Refusal to take part in clinical trial Child C liver cirrhosis Not fit for general anesthesia	Changes in tumor features up to 36 months (size and volume before and after intervention), post procedure adverse events and complication during hospital stay, usually 3 days	
The HistoSonics Edison™ System for Treatment of Primary Solid Renal Tumors Using Histotripsy (#HOPE4KIDNEY) (#HOPE4KIDNEY)	Recruiting	2030-05-01	HistoSonics, Inc.	NCT05820087	primary solid renal tumors	68	Subject is ≥22 years of age. Subject has signed the Institutional Review Board (IRB) approved trial Informed Consent Form (ICF) prior to any trial related tests/procedures and is willing to comply with trial procedures and required follow-up assessments. Subject is diagnosed with only one (1) non-metastatic solid renal mass ≤ 3 cm confirmed via CT or MRI ≤ 30 days prior to the index procedure date. Subject has had a biopsy to determine the type of tumor, ≥14 days prior to the index procedure. Subject can tolerate general anesthesia. Subject has an Eastern Cooperative Oncology Group Performance Status (ECOG PS) grade 0–2 at baseline screening. Subject meets all the following functional criteria at ≤14 days prior to the planned index procedure date: White Blood Count (WBC) ≥ 3000/mm^3^ (≥3 × 10^9^/L) Absolute Neutrophil Count (ANC) ≥ 1200/mm^3^ (≥1.2 × 10^9^/L) Hemoglobin (Hgb) ≥ 9 g/dL Platelet count ≥ 100,000/mm^3^ (≥100 × 10^9^/L) Subject has an eGFR (Glomerular filtration rate) ≥45 mL/min, ≤14 days prior to the planned index procedure date. The tumor selected for histotripsy treatment must be ≤3 cm in longest diameter. Subject has an adequate acoustic window to visualize targeted tumor using the HistoSonics Edison System.	Subject is pregnant or planning to become pregnant or nursing (lactating) during the trial period. Subject is being actively treated in another pharmaceutical or device trial ≤ 30 days prior to planned index procedure date that may interfere with the primary endpoint(s). Subjects who have active cancers (not in remission for the last two years) other than non-melanomatous skin cancers. In the Investigator’s opinion, the subject has co-morbid disease(s) or condition(s) that would cause undue risk and preclude safe use of the HistoSonics Edison System. Subject is on dialysis, being considered for dialysis or has acute renal failure. Subject has not recovered to Common Terminology Criteria for Adverse Events (CTCAE) grade 2 or better from any adverse effects (except alopecia and neuropathy) related to previous therapy. Subject has an International normalized ratio (INR) > 1.5 or uncorrectable coagulopathy (e.g., known von Willebrand disease, hemophilia, or on anticoagulants), on the planned index procedure date. Subject is taking Aspirin (ASA) or NSAIDS ≤ 7 days prior to the planned index procedure date. Subject has a life expectancy less than one (<1) year. In the investigator’s opinion, histotripsy is not a treatment option for the subject. Subject has a concurrent condition that could jeopardize the safety of the subject or compliance with the protocol. Subject’s targeted tumor has had prior locoregional therapy (e.g., ablation, embolization, radiation). Subject’s targeted tumor is not treatable by the HistoSonics Edison System’s working ranges (refer to User Guide). In the investigator’s opinion, the anticipated risks of intervention outweigh the potential benefits of the intervention. Subject has bilateral kidney tumors or has a single functioning kidney. Subject has a genetic predisposition to kidney cancer such as: Von Hippel Lindau (VHL), Hereditary Papillary Renal Carcinoma (HPRC), Birt-Hogg-Dubé Syndrome (BHD), Tuberous Sclerosis Complex (TSC), Hereditary Leiomyomata’s Renal Cell Carcinoma (HLRCC), Reed’s Syndrome, Succinate Dehydrogenase B Deficiency (SDHB), BRCA 1 associated protein -1 (BAP1) Renal Cell Carcinoma, MITF predisposed Renal Cell Carcinoma The targeted tumor is an angiomyolipoma. Subject has a known sensitivity to contrast media and cannot be adequately pre-medicated. Subject has a urinary tract infection (UTI) ≤7 days prior to the planned index procedure date. The targeted tumor is not clearly visible with ultrasound, MRI or CT. Targeted tumor with adequate margin overlaps the renal pelvis, main renal vessel, ureter, organ or other vital structure. The treatment of the tumor will not allow an adequate margin (as determined by the investigator).	Primary technique efficacy defined as the percentage of targeted tumors that were successfully eliminated after a single histotripsy session as assessed by contrast enhanced MRI or CT at 90 days. Primary Safety Endpoint–Freedom from index procedure related major complications, defined by Clavien–Dindo Classification Grade 3 or higher up to 30 days after the histotripsy procedure.	Technical success demonstrating complete coverage of the targeted tumor as determined post-index procedure (≤36 h) by contrast enhanced MRI or CT in subjects whom treatment was initiated. Secondary Safety Endpoint–Freedom from index procedure related major complications, defined by Clavien–Dindo Classification Grade 3 or higher up to 90 days after the histotripsy procedure.
The HistoSonics Investigational System for Treatment of Primary Solid Renal Tumors Using Histotripsy (CAIN)	Active, not recruiting	2025-06-01	HistoSonics, Inc.	NCT05432232	primary solid renal tumors	20	Subject is ≥18 years of age. Subject has signed the Ethics Committee (EC) approved trial Informed Consent Form (ICF) prior to any trial related tests/procedures and is willing to comply with trial procedures and required follow-up assessments. Subject is diagnosed with a non-metastatic solid renal mass ≤ 3 cm confirmed via CT or MRI ≤ 30 days prior to the index procedure date. Subject can tolerate general anesthesia. Subject has an Eastern Cooperative Oncology Group Performance Status (ECOG PS) grade 0–2 at baseline screening. Subject meets all the following functional criteria at ≤14 days prior to the planned index procedure date: White Blood Cell (WBC) ≥ 3000/mm^3^ Absolute Neutrophil Count (ANC) ≥ 1200/mm^3^ Hemoglobin (Hgb) ≥ 9 g/dL Platelet count ≥ 100,000/mm^3^ (≥100 × 10^9^/L) White Blood Cell (WBC) ≤ 40 cells/µL via urinalysis Albumin ≤ 300,000 mg/L via urinalysis Subject has an eGFR ≥ 45 mL/min, ≤14 days prior to the planned index procedure date. International Normalized Ratio (INR) score of <1.5 If on anticoagulants, other than aspirin or non-steroidal anti-inflammatory drugs, assessment must be performed on the day of the procedure; OR If only on aspirin or non-steroidal anti-inflammatory drugs, assessment must be performed ≤14 days prior to the planned index procedure date; OR If not on anticoagulants, assessment must be performed ≤14 days prior to the planned index procedure date Biopsy is required to determine the type of tumor and must be performed ≥14 days prior to the planned index procedure date. The tumor selected for histotripsy treatment must be ≤3 cm in longest diameter. Subject has an adequate acoustic window to visualize targeted tumor using the HistoSonics Investigational System. Subject will undergo histotripsy treatment of only one (1) tumor during the index procedure, regardless of how many tumors the subject has.	Subject is pregnant or planning to become pregnant or nursing (lactating) during the trial period. Subject is enrolled and being actively treated in another investigational pharmaceutical or device trial ≤ 30 days prior to planned index procedure date. Subject is undergoing active chemotherapy for any cancer ≤ 14 days prior to planned index procedure date. Subject is undergoing active immunotherapy ≤ 40 days prior to planned index procedure date. In the Investigator’s opinion, the subject has co-morbid disease(s) or condition(s) that would cause undue risk and preclude safe use of the HistoSonics Investigational System. Subject is on dialysis or being considered for dialysis. Subject has not recovered to Common Terminology Criteria for Adverse Events (CTCAE) grade 2 or better from any adverse effects (except alopecia and neuropathy) related to previous anti-cancer therapy. Subject has an uncorrectable coagulopathy other than that induced by aspirin or non-steroidal anti-inflammatory drugs. Subject has a planned cancer treatment (e.g., nephrectomy, chemotherapy, immunotherapy, etc.) prior to completion of the 30-day follow-up visit. Subject has had previous treatments with chemotherapy, radiotherapy, or both that have not been discontinued ≥14 days prior to the planned index procedure date and have not recovered (CTCAE grade 2 or better) from related toxicity (exclusive of alopecia and neuropathy). Subject has previous treatment with immunotherapies that has not been discontinued ≥40 days prior to the planned index procedure date and has not recovered from related toxicity (CTCAE grade 2 or better). Subject has a life expectancy less than one (<1) year. In the investigator’s opinion, histotripsy is not a treatment option for the subject. Subject has a concurrent condition that could jeopardize the safety of the subject or compliance with the protocol. Subjects’ targeted tumor has had prior locoregional therapy (e.g., ablation, embolization, radiation). Subjects’ tumor is not treatable by the HistoSonics Investigational System’s working ranges (refer to User Guide). In the physician’s opinion, the anticipated risk of intervention outweighs the potential benefits of the intervention. Subject has acute renal failure. Subject has a genetic predisposition to kidney cancer such as: Subject has a genetic predisposition to kidney cancer such as: Von Hippel Lindau (VHL), Hereditary Papillary Renal Carcinoma (HPRC), Birt-Hogg-Dubé Syndrome (BHD), Tuberous Sclerosis Complex (TSC), Hereditary Leiomyomata’s Renal Cell Carcinoma (HLRCC), Reed’s Syndrome, Succinate Dehydrogenase B Deficiency (SDHB), BRCA 1 associated protein -1 (BAP1) Renal Cell Carcinoma, MITF predisposed Renal Cell Carcinoma Tumor is an angiomyolipoma. Subject has a known sensitivity to contrast media and cannot be adequately pre-medicated. The targeted tumor is not clearly visible with diagnostic ultrasound and either magnetic resonance imaging (MRI) or computerized tomography (CT). Targeted tumor with adequate margin overlaps the renal pelvis, main renal vessel, ureter, or other vital structure. Targeted tumor with adequate margin overlaps a non-targeted tumor visible via imaging. The treatment of the tumor will not allow for an adequate margin as determined by the investigator.	Technical success, defined as complete coverage of the tumor as determined ≤36 h post-index procedure by magnetic resonance imaging (MRI) or computerized tomography (CT). Primary Safety: Freedom from Index Procedure Related Major Complications. Freedom from index procedure related major complications, defined by Clavien–Dindo Classification Grade 3 or higher up to 30 days after the last histotripsy procedure.	Percentage of targeted tumors successfully eradicated post-index procedure assessed via MRI or CT at 90 days post-index procedure without repeat Histotripsy. Technique Efficacy (Secondary) Percentage of targeted tumors successfully eradicated post-index procedure assessed via MRI or CT at 90 days post-index procedure after repeat Histotripsy
Real-world Evaluation of the HistoSonics Edison System for Treatment of Liver Tumors Across Multidisciplinary Users (BOOMBOX: Master Study)	Recruiting	2031-11-01	HistoSonics, Inc.	NCT06486454	primary, metastatic, or benign liver tumors	5000	Subject is ≥22 years of age Subject has signed the Ethics Committee (EC), or Institutional Review Board (IRB) approved study Informed Consent Form (ICF) prior to any study related tests/procedures and is willing to comply with study procedures and required follow-up assessments Subject’s liver tumor(s) can be partially or completely treated with histotripsy	Subject is pregnant or planning to become pregnant or nursing (lactating) during the study period Subject is enrolled in an interventional HistoSonics-sponsored trial Subject has a concurrent condition that, in the investigator’s opinion, could jeopardize the safety of the subject or compliance with the protocol	Histotripsy technical success, defined as completion of histotripsy on the target tumor(s) according to the histotripsy treatment plan, assessed by the treating physician on CT or MR imaging at ≤36 h post-histotripsy treatment procedure. The histotripsy treatment plan will include identification of the intended complete or partial treatment of the tumor(s). The histotripsy treatment zone must provide target tumor coverage greater than or equal to the degree of treatment intended.	
The HistoSonics System for Treatment of Primary and Metastatic Liver Tumors Using Histotripsy (#HOPE4LIVER US)	Active, not recruiting	2026-07-01	HistoSonics, Inc.	NCT04572633	primary or metastatic liver tumors	47	Subject is ≥18 years of age Subject has signed the Ethics Committee (EC) or Institutional Review Board (IRB) approved trial Informed Consent Form (ICF) prior to any trial related tests/procedures and is willing to comply with trial procedures and required follow-up assessments Subject is diagnosed with hepatocellular carcinoma (HCC) or liver metastases (mets) from other primary cancers Subject is able to undergo general anesthesia Subject has a Child-Pugh Score of A or B Subject has an Eastern Cooperative Oncology Group Performance Status (ECOG PS) grade 0–2 at baseline screening Subject meets the following functional criteria, ≤7 days prior to the index-procedure: Liver function: Alanine transaminase (ALT) and Aspartate transaminase (AST) < 2.5× upper limit of normal (ULN) and/or bilirubin < 2.5 ULN, and Renal function: serum creatinine < 2× ULN, and Hematologic function: neutrophil count > 1.0 × 10^9^/L and platelet > 50 × 10^9^/L Subject has an International Normalized Ratio (INR) score of <2.0, ≤7 days prior to the index procedure Subject has not responded to and/or has relapsed and/or is intolerant of other available therapies including locoregional therapies, chemotherapy, immunotherapy and targeted therapies The tumor(s) selected for histotripsy treatment must be ≤3 cm in longest diameter Subject has an adequate acoustic window to visualize targeted tumor(s) using ultrasound imaging Subject has a maximum of three (3) tumors to be treated with histotripsy during the index procedure, regardless of how many tumors the subject has.	Subject is pregnant or planning to become pregnant or nursing (lactating) during the trial period Subject is enrolled in another investigational trial and/or is taking investigational medication and/or has been treated with an investigational device ≤ 30 days prior to planned index procedure date In the Investigator’s opinion, the subject has co-morbid disease(s) or condition(s) that would cause undue risk and preclude safe use of the HistoSonics System Subject has a serum creatinine > 2.0 mg/dL or estimated glomerular filtration rate (EGFR) < 30, unless on dialysis Subject has major surgical procedure or significant traumatic injury ≤ 2 weeks prior to the planned index procedure or not fully recovered (CTCAE grade 1 or better) from side effects/complications of such procedure or trauma Subject has not recovered to common terminology criteria for adverse events (CTCAE) grade 1 or better from any adverse effects (except alopecia, fatigue, nausea, vomiting and peripheral neuropathy) related to previous anti-cancer therapy Subject has a history of, or suspected to have, bleeding disorders that are uncorrectable Subject has coagulopathy that is uncorrectable Subject has a planned cancer treatment (e.g., resection, chemotherapy, etc.) after the planned index-procedure date and prior to completion of the 30-day follow-up visit Subject has previous treatment with bevacizumab that has not been discontinued >40 days prior to the planned index-procedure date Subject has planned bevacizumab treatment prior to completion of the 30-day follow-up visit Subject has previous treatments with chemotherapy and/or radiotherapy that has not been discontinued ≥2 weeks prior to the planned index-procedure date and has not recovered (CTCAE grade 1 or better) from related toxicity (except alopecia and peripheral neuropathy) Subject has previous treatment with immunotherapies that has not been discontinued ≥4 weeks prior to the index-procedure and has not recovered from related toxicity (CTCAE grade 1 or better) Subject has a life expectancy less than six (<6) months In the opinion of the Investigator, histotripsy is not a treatment option for the subject Subject has a concurrent condition that, in the investigator’s opinion, could jeopardize the safety of the subject or compliance with the protocol Subjects’ tumor(s) is not treatable by the System’s working ranges (refer to User Manual) Subject has a known sensitivity to contrast media and cannot be adequately pre-medicated Subjects’ target tumor(s) has/have had prior locoregional therapy (e.g., ablation, embolization, radiation) Subject is eligible for surgical resection Targeted tumor(s) treatment volume overlaps a non-targeted tumor visible via imaging The targeted tumor(s) is not clearly visible with diagnostic ultrasound and computed tomography (CT) or magnetic resonance (MR) imaging The targeted tumor(s) is located in liver segment 1 The Planned Treatment Volume intended to cover the targeted tumor includes or encompasses any portion of the main portal vein, common hepatic duct, common bile duct, gallbladder or stomach/bowel.	Technical success, defined as the treatment volume/treatment dimensions being greater than or equal to the targeted tumor, and with complete tumor coverage, via computed tomography (CT) or magnetic resonance (MR) imaging. [Core Laboratory Adjudicated] Primary efficacy was assessed per tumor with a performance goal of greater than 70%. Primary efficacy was assessed after the first forty (40) consecutive evaluable subjects were enrolled. Evaluable subjects had sufficient CT or MR imaging data to allow the independent core laboratory to evaluate technical success. Procedure-Related Major Complications: Number of index procedure related major complications, including device-related events defined as Common Terminology Criteria for Adverse Events (CTCAE) grade 3 or higher toxicities observed up to 30 days post index-procedure. Primary safety was assessed per participant with a performance goal of less than 25%. Primary safety was assessed on all subjects enrolled, after the first forty (40) consecutive subjects evaluable for technical success were enrolled. Evaluable subjects had sufficient CT or MR imaging data to allow the independent core laboratory to evaluate technical success. Enrollment of 44 total subjects was required to assess forty (40) subjects evaluable for technical success.	Technical success, defined as the treatment volume/treatment dimensions being greater than or equal to the targeted tumor, and with complete tumor coverage, via computed tomography (CT) or magnetic resonance (MR) imaging. Number of index procedures related to major complications, including device-related events defined as Common Terminology Criteria for Adverse Events (CTCAE) grade 3 or higher toxicities observed up to 30 days post index-procedure. Technique efficacy, defined as the lack of a nodular or mass-like area of enhancement within or along the edge of the treatment volume assessed via CT or MR imaging at 30 days post-procedure. Number of adverse events (serious and non-serious) reported within 30 days post-index procedure.
The HistoSonics System for Treatment of Primary and Metastatic Liver Tumors Using Histotripsy (#HOPE4LIVER EU/UK) (#HOPE4LIVER)	Active, not recruiting	2026-07-01	HistoSonics, Inc.	NCT04573881	primary or metastatic liver tumors	24	Subject is ≥18 years of age Subject has signed the Ethics Committee (EC) or Institutional Review Board (IRB) approved trial Informed Consent Form (ICF) prior to any trial related tests/procedures and is willing to comply with trial procedures and required follow-up assessments Subject is diagnosed with hepatocellular carcinoma (HCC) or liver metastases (mets) from other primary cancers Subject is able to undergo general anesthesia Subject has a Child-Pugh Score of A or B (up to B8) Subject has an Eastern Cooperative Oncology Group Performance Status (ECOG PS) grade 0–2 at baseline screening Subject meets the following functional criteria, ≤7 days prior to the index-procedure: Liver function: Alanine transaminase (ALT) and Aspartate transaminase (AST) < 2.5× upper limit of normal (ULN) and bilirubin < 2.5× ULN, and Renal function: serum creatinine < 2× ULN, and Hematologic function: neutrophil count > 1.0 × 10^9^/L and platelet > 50 × 10^9^/L Subject has an International Normalized Ratio (INR) score of <2.0, ≤7 days prior to the index procedure Subject has not responded to and/or has relapsed and/or is intolerant of other available therapies including locoregional therapies, chemotherapy, immunotherapy and targeted therapies. The tumor(s) selected for histotripsy treatment must be ≤3 cm in longest diameter Subject has an adequate acoustic window to visualize targeted tumor(s) using ultrasound imaging Subject has a maximum of three (3) tumors to be treated with histotripsy during the index procedure, regardless of how many tumors the subject has.	Subject is pregnant or planning to become pregnant or nursing (lactating) during the trial period Subject is enrolled in another investigational trial and/or is taking investigational medication or treated with an investigational device ≤ 30 days prior to index procedure In the Investigator’s opinion, the subject has co-morbid disease(s) or condition(s) that would cause undue risk and preclude safe use of the HistoSonics System Subject has a serum creatinine > 2.0 mg/dL or estimated glomerular filtration rate (EGFR) < 30, unless on dialysis Subject has major surgical procedure or significant traumatic injury ≤ 2 weeks prior to the index procedure or not fully recovered from side effects/complications of such procedure or trauma Subject has not recovered to common terminology criteria for adverse events (CTCAE) grade 1 or better from any adverse effects (except alopecia) related to previous anti-cancer therapy Subject has a history of, or suspected to have, bleeding disorders that are uncorrectable Subject has a coagulopathy that is uncorrectable Subject has a planned cancer treatment (e.g., resection, chemotherapy, etc.) from the index-procedure date and prior to completion of the 30 day follow-up visit Subject has previous treatment with bevacizumab that has not been discontinued >40 days prior to the planned index-procedure date Subject has planned bevacizumab treatment prior to completion of the 30 day follow-up visit Subject has previous treatments with chemotherapy and/or radiotherapy that has not been discontinued ≥2 weeks prior to the planned index-procedure date or has not recovered from related toxicity Subject has previous treatment with immunotherapies that has not been discontinued ≥4 weeks prior to the index-procedure or has not recovered from related toxicity Subject has a life expectancy less than six (<6) months In the opinion of the Investigator, histotripsy is not a treatment option for the subject Subject has a concurrent condition that, in the investigator’s opinion, could jeopardize the safety of the subject or compliance with the protocol Subjects’ tumor(s) is not treatable by the System’s working ranges (refer to User Manual) Subject has a known sensitivity to contrast media and cannot be adequately pre-medicated Subjects’ targeted tumor(s) has/have had prior locoregional therapy (e.g., ablation, embolization, radiation) Subject is eligible for surgical resection Targeted tumor(s) treatment volume overlaps a non-targeted tumor visible via imaging The targeted tumor(s) is not clearly visible with diagnostic ultrasound and computed tomography (CT) or magnetic resonance (MR) imaging The targeted tumor(s) is located in liver segment 1 The Planned Treatment Volume intended to cover the targeted tumor includes or encompasses any portion of the main portal vein, common hepatic duct, common bile duct, gallbladder or stomach/bowel	Technical success, defined as the treatment volume/treatment dimensions being greater than or equal to the targeted tumor, and with complete tumor coverage, via computed tomography (CT) or magnetic resonance (MR) imaging. Number of index procedure related major complications, including device-related events defined as Common Terminology Criteria for Adverse Events (CTCAE) grade 3 or higher toxicities observed up to 30 days post index-procedure.	Technique efficacy, defined as the lack of a nodular or mass-like area of enhancement within or along the edge of the treatment volume assessed via CT or MR imaging at 30 days post-procedure. Number of adverse events (serious and non-serious) reported within 30 days post-index procedure
Treatment of Cancer with Immune Checkpoint Inhibition Therapy Boosted by High Intensity Focused Ultrasound Histotripsy	Active, not recruiting	2030-08-01	UMC Utrecht	NCT06524570	metastatic or unresectable cancer	24	Histologically confirmed metastatic or unresectable cancer that progressed under standard of care treatment options. Age ≥ 18 years. Has signed and dated written informed consent before performing any study procedure, including screening. Anticipated life expectancy ≥12 weeks by investigator judgment. At least one tumor lesion (primary tumor or metastasis) which is amenable to application of high intensity focused ultrasound histotripsy (determined by a radiologist with HIFU-expertise). The lesion must have a distance of ≤30 mm to the skin. At least part of the lesion must have a distance of ≥10 mm to the skin and other vulnerable structures (e.g., large blood vessels). This part should be sufficient to be able to select at least one HT focus in an area of solid tumor. If the target lesion contains cystic or necrotic regions: the solid component should be ≥10 mm in diameter, sufficient to be able to select at least one HIFU-HT focus in an area of solid tumor with ≥10 mm distance to the skin. Sonication will be performed on tumors that have not previously directly been treated with radiation therapy or surgery unless they showed significant mass regrowth. Measurable disease (at least one lesion besides the HIFU-HT treated lesion) on CT according to RECIST V 1.1 criteria (or on PET-CT according to PERCIST criteria) as assessed by investigator and local radiology review. Performance status of 0 or 1 on the WHO Performance Scale. Screening laboratory values must meet the following criteria: WBC ≥ 2.0 × 10^9^/L, Neutrophils ≥ 1.5 × 10^9^/L Platelets ≥ 100 × 10^9^/L Hemoglobin ≥ 5.5 mmol/L Serum creatinine ≤ 1.5× upper limit of normal (ULN) or calculated creatinine clearance ≥ 60 mL/min (≤Grade 1) Aspartate aminotransferase (AST) ≤ 2.5× ULN; alanine aminotransferase (ALT) ≤ 2.5× ULN; AST/ALT < 5× ULN if liver involvement Serum bilirubin ≤ 1.5× ULN or direct bilirubin ≤ ULN for subjects with total bilirubin levels > 1.5× ULN, except in subjects with Gilbert’s Syndrome Patients must agree to use an adequate method of contraception for the course of the study through 180 days after the last dose of study medication. Patients must be willing to undergo tumor biopsy.	Presence of known central nervous system, meningeal, or epidural metastatic disease. However, subjects with known brain metastases are allowed if the brain metastases are stable for ≥4 weeks before the first dose of study treatment. Stable is defined as neurological symptoms not present or resolved to baseline, no radiologic evidence of progression, and steroid requirement of prednisone ≤10 mg/day or equivalent. Patients currently participating and receiving study therapy or patients who participated in a study of an investigational agent and received study therapy or used an investigational device within 4 weeks prior to the first dose of the study treatment. Prior chemotherapy, targeted small molecule therapy or monoclonal antibodies within 4 weeks prior to the first dose of the study treatment. Prior radiotherapy within 8 weeks prior to the first dose of the study treatment. The patient will be excluded from the study if the only targetable lesion has directly been treated with radiation therapy in the past with an exception for lesions that showed massive regrowth. Prior surgery or ablative therapy within 4 weeks prior to the first dose of the study treatment. The patient will be excluded from the study if the only targetable lesion has directly been treated with ablative therapy in the past. Ongoing adverse events > Grade 1 due to a previously administered therapy. Subjects with ≤Grade 2 neuropathy, vitiligo, thyroid disorders, hypocortisolism or alopecia of any grade are an exception to this criterion and may qualify for the study. History of other malignancies, except adequately treated and a cancer-related life-expectancy of more than 5 years. Concurrent medical condition requiring the use of immunosuppressive medications, or immunosuppressive doses of systemic or absorbable topical corticosteroids; exceeding prednisolone 10 mg or equivalent. Active autoimmune disease that has required systemic treatment in the past 2 years (i.e., with use of disease modifying agents, high-dose corticosteroids or immunosuppressive drugs). Replacement therapy (e.g., thyroxine, insulin, or physiologic corticosteroid replacement therapy for adrenal or pituitary insufficiency, etc.) is not considered a form of systemic treatment. Active infection requiring systemic therapy. History of (non-infectious) pneumonitis that required steroids or current pneumonitis. Known history of active Tuberculosis. Receipt of a live vaccine within 4 weeks prior to the first dose of the study treatment. Hypersensitivity to any of the study drugs or their excipients. Contra-indications to MR imaging (e.g., certain pacemakers or severe claustrophobia). Contra-indications to gadolinium-based contrast agents are not an exclusion criterion, as a different brand of gadolinium can be used or if necessary the MRI can be performed without contrast. Pregnancy or lactation. Any other medical or social condition that, in the opinion of the Principal Investigator, might put the subject at risk of harm during the study or might adversely affect the interpretation of the study data.	Number and severity of adverse events until 100 days after the last study treatment Discontinuation rate due to adverse events at every visit until 2 years post treatment Patient reported tolerability by HIFU-HT-tolerability questionnaire: The HIFU-HT tolerability questionnaire is a self-reported, customized questionnaire that describes the burden/complaints a respondent experienced following HIFU-histotripsy treatment. The questionnaire comprises questions about pain, use of pain medication, complaints other than pain, burden of MRI scan, burden of peri-procedural analgesia, time burden of treatment. Respondents are asked to grade the experienced complaints or burden on a scale of 5 options, ranging from no complaints/no burden to severe complaints/severe burden. If respondents report pain, they are asked to grade their pain on a scale ranging from 0 to 10 (0 reflecting no pain, 10 reflecting worst possible pain) and respondents are asked for how many days the pain was present (ranging from 0 to 7 days). This will be performed at days 8 and 15. Patient reported tolerability by EQ-5D: The EuroQol Group EQ-5D questionnaire (Dutch version) is a self-reported questionnaire that reflects a respondent’s health. The EQ-5D comprises questions on 5 domains (mobility, self care, daily activities, pain/complaints, mood), for each of these domains respondents state whether they have no problems, some problems or severe problems. Respondents are also asked to grade their general health status on a scale of 0–100 (0 reflecting the worst possible health status, 100 reflecting the best possible health status). This will be performed at baseline, days 1, 8, 15, 22, 43, 64, 91; thereafter every 4 to 8 weeks until 2 years after start of therapy Patient reported tolerability by USD-I: The Utrecht symptom diary immunotherapy (USD-I) is a self-reported questionnaire that was developed and validated in the UMC Utrecht to score symptoms patients might experience during/after treatment with checkpoint inhibition therapy. The questionnaire comprise questions on 19 possible symptoms (apetite, stool pattern, diarrhea, abdominal pain, coughing, eye complaints, skin rash, pruritus, headache, myalgia, arthralgia, paresthesias, pain, sleeping problems). Respondents are asked to grade these symptoms on a scale of 0–10 (0 reflecting no problems, 10 reflecting worst possible problem). This will be performed at baseline, days 1, 8, 15, 22, 43, 64, 91; thereafter every 4 weeks until 2 years after start of therapy Feasibility: Number of technically effective HIFU-HT procedures on day 8, percentage of screening failures at baseline, and time burden of the study procedures through study completion up to two years after start of study treatment	Radiological response: MRI- Local response of HIFU-HT treated tumor as assessed by MRI directly and 12 weeks after HIFU-HT Radiological response: CT—Best overall systemic response using RECIST 1.1 as assessed by CT-scan every 12 weeks (or using PERCIST as assessed by PET-CT if not RECIST measurable) Immunologic response: Analysis of immunological parameters in peripheral blood. Analysis of immune infiltrates in tumor biopsies taken at baseline and 7 days after HIFU-HT-Baseline and days 1, 8, 9, 15, 22, 64 Overall survival: Explorative analysis to assess overall survival in months while taking into consideration the heterogeneous patient population in this basket design. Performed every 12 weeks until 2 years. Progression free survival: Explorative analysis to assess progression-free survival in months while taking into consideration the heterogeneous patient population in this basket design. Done every 12 weeks until 2 years.
The HistoSonics Edison™ System for Treatment of Pancreatic Adenocarcinoma Using Histotripsy	Recruiting	2026-01-01	HistoSonics, Inc.	NCT06282809	pancreatic adenocarcinoma	50	Subject is ≥18 years of age. Subject has signed the Ethics Committee (EC) approved trial Informed Consent Form (ICF) prior to any trial related tests/procedures and is willing to comply with trial procedures and required follow-up assessments. Subject is diagnosed with unresectable pancreatic adenocarcinoma, locally advanced (Stage 3) or oligometastatic disease (Stage 4) confirmed via CT or MR imaging ≤30 days prior to the index procedure date. NOTE: If Stage 4 disease, there must be ≤5 metastatic tumors and the tumors are located only in the liver and/or lung. Subject is not a surgical candidate and has received chemotherapy ≥8 weeks. Subject can tolerate general anesthesia. Subject has an Eastern Cooperative Oncology Group Performance Status (ECOG PS) grade 0–1 at baseline. Subject meets the following criteria ≤14 days prior to the planned index procedure date: Hemoglobin ≥ 9 g/dL, Neutrophil count > 1.0 × 10^9^/L, Platelet > 50 × 10^9^/L, Total bilirubin ≤ 2.5× Institutional Upper Limit of Normal (IULN), Aspartate aminotransferase (AST) and Alanine aminotransferase (ALT) ≤2.5× IULN, International Normalized Ratio (INR) value <1.5, Serum creatinine < 2.0 mg/dL or an estimated glomerular filtration rate (eGFR) ≥45 mL/min. The targeted pancreatic tumor is ≥2 cm in longest diameter. The planned histotripsy treatment volume is ≥1.0 cm from any portion of the duodenum, small intestine, stomach, or colon as visualized on ultrasound, and CT, or MR imaging. Subject has an adequate acoustic window to visualize targeted tumor using the HistoSonics Edison System. Subject will undergo histotripsy treatment of only one (1) tumor during the index procedure, regardless of how many tumors are present in the pancreas.	Subject is pregnant or planning to become pregnant or nursing (lactating) during the trial period. Subject has had prior pancreatic, bilioenteric, or gastric surgery. Subject is being actively treated in another pharmaceutical or device trial that has not completed its primary endpoint prior to the index procedure or may interfere with the primary outcome measure of this trial. Subject has an uncorrectable coagulopathy. Subject has a life expectancy of less than six (6) months. Subject has a biliary or pancreatic stent and/or percutaneous biliary tube that encompasses the planned histotripsy treatment volume. Subject has metastases to organs other than the liver and/or lung (e.g., bone, brain, peritoneum). Subject has a known sensitivity to contrast media and cannot be adequately pre-medicated. Subject has an active duodenal or gastric ulcer requiring medical management. Subject is undergoing active chemotherapy for any cancer ≤ 14 days prior to planned index procedure date. Subject is undergoing active immunotherapy ≤ 30 days prior to planned index procedure date. Subject’s targeted tumor has had prior locoregional therapy (e.g., ablation, embolization, or radiation). Subject has a planned cancer treatment (e.g., pancreatic surgery, chemotherapy, immunotherapy, etc.) prior to completion of the 30 day follow-up visit. Subject has not recovered (CTCAE grade 2 or better) from chemotherapy or immunotherapy related toxicities (exclusive of alopecia, neuropathy, and exocrine insufficiency). In the investigator’s opinion, histotripsy is not a treatment option for the subject. Subject has a concurrent condition that could jeopardize the safety of the subject or compliance with the protocol. Subject’s tumor is not treatable by the System’s working ranges (refer to User Guide).	Evaluate the safety of the HistoSonics Edison System for the destruction of pancreatic adenocarcinomas using histotripsy: Index procedure-related complications ≤ 30 days post index procedure, graded using Clavien–Dindo Classification and Common Terminology Criteria for Adverse Events (CTCAE).	

## Abbreviations

FDA—United States Food and Drug Administration; CD—Cluster of Differentiation; HCC—Hepatocellular Carcinoma; ICC—Intrahepatic Cholangiocarcinoma; PAC—Pancreatic Adenocarcinoma; RCC—Renal Cell Carcinoma; HIFU—High Intensity Focused Ultrasound; MPa—Mega Pascals; ECM—Extracellular Matrix; PRF—Pulse Repetition Frequency; FWHM—Full Width Half Maximum; MRI—Magnetic Resonance Imaging; IFN—Interferon; HER2—Human Epidermal Growth Factor 2; CT—Computed Tomography; VX2—anaplastic squamous cell carcinoma animal model derived from Shope papillomavirus infection and used by interventional radiologists; TNF—Tumor Necrosis Factor; IL—Interleukin; HIF—Hypoxia Inducible Factor; CXCL—C-X-C Chemokine Ligand; CXCR—C-X-C Chemokine Receptor; MEK—Mitogen-Activated Extracellular Signal-Regulated Kinase; US—Ultrasound; NPO—nil per os (nothing by mouth); MHz—Mega-Hertz; DOF—Degrees Of Freedom; B-mode—Brightness mode; ARFI—Acoustic Radiation Force Imaging; PD-L1—Programmed Death—Ligand 1; CTLA4—Cytotoxic T Lymphocyte Antigen 4.
